# CT imaging for occult malignancy in patients with unprovoked venous thromboembolism (VTE) in a tertiary centre: is it worthwhile?

**DOI:** 10.1007/s11845-023-03317-6

**Published:** 2023-02-27

**Authors:** Ronan J. Lee, Darragh Herlihy, Damien C. O’Neill, Lauren Madden-Doyle, Martina Morrin, Michael J. Lee

**Affiliations:** Beaumont Hospital, Beaumont Road, Dublin 9, Dublin, Ireland

**Keywords:** Deep venous thrombosis, Occult malignancy, Pulmonary embolism, Unprovoked venous thromboembolism

## Abstract

**Background:**

Investigating patients with unprovoked venous thromboembolism (uVTE) for occult malignancy can prove a diagnostic dilemma and imaging is often used extensively in this patient group.

**Aims:**

The primary objective of this study was to determine the incidence of malignancy on CT and other imaging over a 10-year period. A secondary objective was to evaluate the role of laboratory and other non-imaging tests performed.

**Methods:**

A retrospective key word search of our hospital’s imaging system was performed to identify patients with unprovoked DVT/PE over the last 10 years. All imaging, histology, endoscopy, laboratory tests, and clinical follow-up over 2 years were analysed. Patients with provoked VTE were excluded.

**Results:**

150 patients had uVTE. 9 patients were diagnosed with occult malignancy by different investigations on index hospital admission (3 patients) or subsequently on clinical follow-up (6 patients). Mean age of patients was 62 years. 116 patients had CT body imaging. The incidence of malignancy diagnosed by initial CT imaging was 1.7% with a sensitivity of 22%, specificity 87%, and PPV 12.5%. Overall incidence of malignancy identified by imaging alone during the index hospital admission was 2%. Total incidence of malignancy including index admission and follow-up was 6%. Median time to cancer diagnosis was 12 months.

**Conclusion:**

CT imaging had a low yield for diagnosing malignancy. Extensive imaging strategies increase cost and radiation exposure without improving mortality. Clinical follow-up, history taking, and physical examination guiding appropriate investigations remain the best tool for unmasking occult malignancy in patients with uVTE.

## Introduction

Unprovoked venous thromboembolism (uVTE) can pose a diagnostic dilemma for physicians as it can be associated with underlying occult malignancy or thrombophilia. Malignancy is a well-established independent risk factor for VTE. Incidence of malignancy associated with unprovoked VTE ranges from 4 to 10% [1, 2], and the chance of discovering occult malignancy is higher in the first 6–12 months after uVTE [3–5]. There is frequently a heterogenous approach to investigating unprovoked VTE in practise. The 2 main approaches to investigating occult cancer in uVTE are (1) a limited investigative approach which involves obtaining basic laboratory tests (including full blood count, serum calcium, urinalysis, and liver function tests), a chest radiograph in addition to targeted cancer screening based on age, gender, history, and physical exam; or (2) an extensive investigative approach composed of laboratory investigations and cross-sectional imaging in the form of computed tomography (CT), positron emission tomography (PET/CT), and ultrasound [6]. It is unclear how aggressively physicians should be screening for occult cancer in this scenario. Recent NICE guidelines (2020) recommend a limited investigative approach in the setting of occult malignancy screening in uVTE. A comprehensive imaging approach should only be considered in addition to a limited investigative approach if a patient has relevant signs and symptoms of malignancy that would make a physician suspect underlying cancer such as unexplained weight loss, bone pain, night sweats, or a new abdominal mass for example [7]. The primary objective of this study was to ascertain the incidence of occult malignancy in a cohort of patients with unprovoked DVT/PE in our tertiary referral centre who underwent CT examinations of thorax, abdomen, and pelvis as well as other imaging tests performed. A secondary objective was to evaluate the role of laboratory and other non-imaging tests performed.

## Materials and methods

We performed a retrospective search of our local hospital picture and archiving system (PACS) over a 10-year period (2010–2020) to identify patients with unprovoked DVT/PE who had imaging performed. Search terms included “unprovoked PE”, “unprovoked pulmonary embolism”, and “unprovoked DVT”.

The exclusion criteria included provoked VTE at the time of imaging and patients who were later diagnosed with provoked VTE in a haematology clinic. Three of the authors were involved in the data collection.

Laboratory tests including histology and blood markers (FBC, calcium, LFTs, LDH, serum protein electrophoresis (SPEP), thrombophilia screening, and tumour markers) were reviewed. Endoscopy (including upper gastrointestinal endoscopy and colonoscopy) and imaging tests such as chest radiograph, CT, PET/CT, and ultrasound were also recorded. Clinic letters and imaging were assessed for all patients over a 24-month period follow-up period to assess if any malignancies or cause for unprovoked VTE could be identified on follow-up assessment. We followed these patients over a 2-year period to establish if any patient developed malignancy or a thrombophilia within that time frame.

This study was approved by our hospitals department of clinical audit and governance.

## Results

Our search of PACS identified 154 patients with uVTE. 4 patients were excluded as they were diagnosed with provoked VTE in haematology clinic follow up. We therefore identified 150 eligible patients (107 PE, 36 DVT, and 7 PE/DVT) who had been investigated for unprovoked VTE. Ultimately, 9 patients were diagnosed with occult malignancy by different investigations and at different time points either on the index hospital admission (3 patients) or subsequently on clinical follow-up (6 patients). Mean age patients was 62 years (median 64). 117 patients were over the age of 50 (78%) (Table 1). 80 patients were between the ages of 50 and 74 and 37 patients were 75 or older (Table 1). 79 patients were female and 71 were male with a predominance of cancer identified in females (7) versus males (2) (Table 2).

**Table 1:** Age profile of patients

**Age**	**Malignancy identified**	**Patients**
**< 50**	2 (1.3%)	33
**50–74**	5 (3.3%)	80
**75 + **	2 (1.3%)	37

**Table 2:** Gender profile of patients

**Gender**	**Malignancies by gender**
**M (71)**	2
**F (79)**	7

### Index hospital admission imaging and laboratory test results

116 patients had CT body imaging. 18/116 patients had a CT TAP only. 20/116 patients underwent a CTPA followed by CT TAP. 77/116 patients had a CTPA followed by a CT abdomen pelvis (CTPA did not include lung apices). 1 patient had a CT abdomen with chest radiograph only. 16 of the 116 patients who had CT imaging were identified as having a possible malignancy. These 16 patients were investigated and discussed at multi-disciplinary team meetings (MDT) (Table 3). 2 of these patients were diagnosed on their initial CT with malignancy (breast and bladder cancer). The patient with breast malignancy was started on hormonal treatment due to her advanced age and is still alive 2 years later. The patient with bladder malignancy was noted to have hydronephrosis on their CTPA and proceeded to have a completion CT abdomen and pelvis, which showed a mass in the bladder. This was biopsied and a diagnosis of bladder cancer was made. However, this patient died of sepsis 2 months later after a trans-urethral resection of bladder tumour (Table 4). The incidence of malignancy diagnosed by initial CT imaging was 1.7% (2/116) with a sensitivity of 22%, specificity 87%, and PPV 12.5%. The remaining 14/16 patients were investigated for the abnormality detected on CT but no malignancy was found (Table 3).

**Table 3:** Possible malignancies identified with CT and the outcome

**Possible malignancy on initial CT-TAP**	**Investigation and comments**	**Outcome**
**Bladder mass**	Biopsy	Malignant
**Parotid mass**	Biopsy	Benign Warthin tumour
**Renal mass**	Cystoscopy histology showed benign	Benign complex renal cyst
**Renal lesion**	US	Benign kidney cyst
**Adrenal lesion**	CT TAP	Adrenal adenoma
**Uterine mass**	Mammogram separate and biopsy	Malignant DCIS, benign uterine fibroid
**Breast lesion**	Biopsy	Benign fibroadenoma
**Breast lesion**	Biopsy	Malignant-incidental
**Rectal thickening**	Colonoscopy and biopsy	Normal rectal tissue
**Lung nodule**	2 interval CT thorax	Benign lung lesion
**Lung nodule**	Resolution of lung nodules	Benign
**Nerve sheath tumour**	Reviewed	Artefact
**Omental mass**	2 interval CT TAP and PET CT	Benign omental mass
**Pancreatic lesion**	Follow-up CT showed no lesion	Benign
**LIF mass**	PET and CT showed interval reduction in size	Benign LIF mass
**Pelvic mass**	Taking a DOAC	Rectus sheath haematoma

**Table 4:** Malignancies identified on index admission with demographics and method of detection

**Malignancy**	**Age**	**Gender**	**Detected by?**	**Limited or extensive strategy?**	**Time to diagnosis in months**
**Endometrial**	69	f	Ultrasound (no CT undertaken)	Extensive	0
**Bladder**	86	f	CT-TAP	Extensive	0
**Breast**	90	f	CT-TAP	Extensive	0

In terms of other imaging, 5 patients diagnosed with unprovoked DVT had ultrasound imaging without having undergone CT imaging. Endometrial cancer was identified on a pelvic ultrasound in one of these patients who had active post-menopausal bleeding on their index hospital admission. 137 of the 150 (91%) patients had a chest radiograph during their index hospital admission. 2 patients had lung nodules on chest x-ray. These lung nodules were found to be spurious on follow-up CT. Overall incidence of malignancy identified on imaging alone during the index hospital admission was 2% (3/150).

With regard to laboratory tests, all patients had an array of blood tests based on clinical discretion during their hospital admission, which aided further investigations. Standard blood tests performed during the index hospital admission included a full blood count (FBC), white cell count (WCC), corrected calcium, and liver function tests (LFT). With regard to FBC, 27 patients had anaemia and 2 (1.3%) patients were subsequently diagnosed with malignancy (lung (1) and endometrial cancer (1)). 3 patients had polycythaemia. 8 patients had thrombocytopaenia. 3 patients had thrombocytosis with no malignancy identified. When analysing the WCC, 10 patients had leucocytosis. 9 patients’ leucocytosis resolved during their hospital admission and 1 (0.7%) patient was diagnosed with chronic eosinophilic leukaemia 2 months later. 131 patients had liver function tests (LFTs) performed. 44 patients had normal LFTs. 87 patients had mildly deranged LFTs. 3 patients with mildly deranged LFTs were subsequently found to have malignancy (non-metastatic colorectal cancer (1), chronic eosinophilic leukaemia (1), and bladder cancer (1)).

### Follow-up after index admission

126 (79%) patients had follow-up in an outpatient clinic (15 month mean time of follow-up, 24 month median time of follow-up, and range 1 month to 24 months) with 11 patients having a repeat CT TAP in the follow-up period with one CT suspicious for lung cancer, which was investigated with PET-CT. In total, 2 patients had PET-CT imaging: 1 patient had a possible left iliac fossa mass on follow-up CT, which resolved and did not require follow-up, and 1 patient had a lung lesion on a follow-up CT thorax, which PET-CT followed by lung biopsy confirmed to be a lung cancer.

Of the 133 patients who had clinical follow-up, we identified 6 patients with malignancy within the 24-month period. These included 5 solid malignancies and 1 haematological malignancy: colorectal (1), breast (1), prostate (1), glioblastoma multiforme (GBM) (1), lung (1), and chronic eosinophilic leukaemia (1) with an average time to diagnosis of 11 months. Four of these patients were over the age of 50 and 2 patients under the age of 50 (Table 5). These patients had their initial CT-TAP reassessed and no malignancies were identifiable on their initial imaging. Of the 7 malignancies not identified by CT-TAP, 2 malignancies were identified by laboratory tests (chronic eosinophilic leukaemia and prostate cancer) and 1 by endoscopy whilst the remaining malignancies were identified on ultrasound, CT brain, and mammography (Table 5). 14 patients had a mammogram with 2 breast cancers identified.

**Table 5:** Malignancies identified on follow-up with demographics and method of detection

**Malignancy**	**Age**	**Gender**	**Initial investigation which detected malignancy**	**Limited or extensive strategy**	**Time to diagnosis in months**
**Chronic eosinophilic leukaemia**	30	m	Laboratory investigations	Extensive	2
**Breast**	41	F	Mammogram	Extensive	1
**Colorectal**	69	F	Endoscopy	Extensive	18
**Prostate**	68	m	Laboratory investigations	Extensive	9
**GBM**	69	f	CT brain	Extensive	15
**Lung**	69	f	CT-TAP	Extensive	22

Tumour markers were performed for 62 (41.3%) patients in total. Tumour markers performed included PSA, CA-125, AFP, B-HCG, and CA 19.9. Patient gender and age influenced the selection of tumour markers. 2 of the 9 patients with malignancy had tumour markers performed. A patient with prostate cancer (0.7%) had an elevated PSA of 4.5 and a patient with breast cancer (0.7%) had a negative CA-125. There were 7 positive PSA tests in total (ranging from 4.4 to 8.1) with one malignancy identified. One patient had a PSA of 4.5, which was repeated and was stable but did not receive any follow-up or imaging. The urology team followed the remaining 5 patients with PSA surveillance and benign prostatic hyperplasia (BPH) was diagnosed in these 5 patients. One patient had a PI-RADS 3 lesion on MR imaging but declined a biopsy and is currently undergoing PSA surveillance.

There were 86 thrombophilia screens performed with 1 diagnosis of essential thrombocytosis. Lactate dehydrogenase (LDH) was performed in 25 patients and an elevated LDH was identified in 15 patients. 1 patient with an elevated LDH was diagnosed with chronic eosinophilic leukaemia. 56 serum protein electrophoresis (SPEP) tests were performed with 2 patients diagnosed with monoclonal gammopathy of undetermined significance (MGUS).

Oesophago-gastro-duodenoscopy (OGD) and colonoscopy were performed in 24 patients during follow-up. One colonoscopy prompted by weight loss demonstrated a sigmoid colon cancer which was confirmed by histology. On review, this was not visible on CT imaging.

## Discussion

Several studies show that about 90% of occult malignancies can be diagnosed by a limited investigative approach with little difference in mortality between the limited and extensive investigative strategies [2, 8–10]. Tumour markers alone have been shown to be an inadequate screening tool for malignancy in the general population [11]. The strategy of extensive comprehensive investigation is associated with more false positives with increased patient anxiety and higher cost [12].

On analysis of the literature and our patient group, it is clear that investigation of patients with uVTE causes uncertainty for physicians. There is heterogeneity regarding the investigative approach for occult malignancy in uVTE in our patient group over the last 10 years. On review of the 137 chest x-ray requests, uVTE was not mentioned in the clinical information provided in 91% of requests demonstrating a potential unawareness of NICE guidelines. Laboratory tests also had a myriad of combinations of basic blood tests and tumour markers, thrombophilia screens. Extensive imaging was favoured in our centre with 77% of patients receiving at least one CT, which points to an overreliance on computed tomography.

Total incidence of occult cancer in uVTE ranges from 4 to 10% in the literature [1–4, 9, 10]; however, a recently published systematic review [1] and 2 recently published randomised trials (SOME [2] MVTEP [4]) showed a lower incidence of occult malignancy in approximately 4–6% of patients with uVTE; however, the reason why remains unclear. The total incidence of malignancy was 6% (index admission and follow-up) in our patient cohort, which is in line with the current literature [1, 2, 4, 13, 14]. Median time to cancer diagnosis was 12 months with 6 malignancies (4%) diagnosed at 1 year, and 3 malignancies (2%) identified at 24 months signifying the importance of continued clinical follow-up of these patients. 78% of patients in our study diagnosed with cancer were over the age of 50 signifying increasing age as an important risk factor [2, 4]. There was additionally a predilection for malignancy in the female population (78%), which is contrary to the Robin et al. study (which compared a limited screening to PET/CT screening approach) [4], which showed a predilection for male gender. So, this finding of a predilection of occult malignancy in female gender may be spurious due to the lower power of this study.

We found that CT imaging had a low yield for diagnosing malignancy (1.7%), which echoes findings in the literature with an incidence of 2–2.3% [13, 14]. CT had a sensitivity of 22%, specificity 87%, and PPV 12.5%. No other studies comment on overall CT sensitivity/specificity. Additionally, the malignancies encountered, such as organ confined prostate cancer, breast cancer, and most early-stage cancers including colorectal cancer, would generally not be identified on CT.

CT scanning of the abdomen and pelvis has been shown to be both more costly without being more effective in detecting occult cancer [12, 15]. It is well established in the literature that the addition of CT-TAP to a limited strategy does not reduce cancer-related mortality [2, 16]. Thus, the effects of radiation exposure must be considered when determining the utility of CT as a mechanism for cancer detection in this patient population. Indeed, most studies identified, including NICE guidelines, conclude that the addition of CT to a limited investigative approach is generally not warranted [2, 7, 10, 12, 16]. With regard to PET-CT, our centre used external PET imaging for only 2 patients and 1 patient was diagnosed with lung cancer having a high index of suspicion based on an enlarging lung nodule. It should be noted that other studies did not have much success with a PET-CT strategy with PET-CT not offering a higher diagnosis accuracy than CT [4, 17]. However, there are ongoing trials in this area specifically the MVTEP/SOME 2 trial, which is a randomised control trial assessing limited screening investigations versus PET/CT screening in patients with uVTE over 50 years of age, which may further clarify the usefulness of PET/CT imaging in uVTE [18]. Additionally, of interest, there is another study ongoing called the PLATO-VTE trial which is assessing the role of novel biomarkers to detect occult cancer which may prove to be a method of detecting occult malignancy without the use of radiation [18].

Whilst a small number of patients were diagnosed with haematological disease, we believe thrombophilia screening should be used with the advice of the haematology department in accordance with NICE guidelines as they can be difficult to interpret without specialist input.

Laboratory investigation as per NICE guidelines proved to be helpful in unmasking underlying malignancy in our series. 3 malignancies of the 9 (33%) were identified on laboratory testing. However, interpreting these tests during an acute index admission retrospectively has limitations as it is highly dependent on the clinical context at the time. We believe that a careful follow-up clinic strategy with judicious use of imaging following NICE guidelines is warranted.

We recognise some limitations to our study in that the study was retrospective and had a somewhat lower sample size, and our key word search may not have identified all patients with uVTE. In addition, not all patients had a complete CT TAP, which may have introduced bias in terms of lesion being missed.

## Conclusion

The original 2012 NICE guidelines prompted an unintended increase in the use of CT for detecting occult malignancy in unprovoked VTE [7]. Recent NICE guidelines 2020 have reiterated that extensive imaging should only be instituted for patients based on a strong foundation of history and examination eliciting the appropriate signs and symptoms [7]. In our study, CT imaging had a low yield for diagnosing malignancy (1.7%), and we feel that its ubiquitous use may not be the most effective tool to diagnosis occult malignancy in this context. Extensive imaging strategies increase cost and radiation exposure. Clinical follow-up, history taking, and physical examination guiding appropriate investigations remain the best tool for unmasking occult malignancy in patients with unprovoked venous thromboembolism.

## Data Availability

The data that support the findings of this study are available on reasonable request from the corresponding author. The raw data are not publicly available due to GDPR restrictions.
